# Maternal sensitivity and infant neural response to touch: an fNIRS study

**DOI:** 10.1093/scan/nsab069

**Published:** 2021-06-18

**Authors:** Vera Mateus, Ana Osório, Helga O Miguel, Sara Cruz, Adriana Sampaio

**Affiliations:** Developmental Disorders Graduate Program, Center for Biological and Health Sciences, Mackenzie Presbyterian University, São Paulo 01302-000, Brazil; Developmental Disorders Graduate Program, Center for Biological and Health Sciences, Mackenzie Presbyterian University, São Paulo 01302-000, Brazil; Psychological Neuroscience Laboratory, CIPsi, School of Psychology, University of Minho, Braga 4710-057, Portugal; The Psychology for Positive Development Research Center, Lusíada University – North, Porto 4369-006, Portugal; Psychological Neuroscience Laboratory, CIPsi, School of Psychology, University of Minho, Braga 4710-057, Portugal

**Keywords:** fNIRS, maternal sensitivity, somatosensory cortex, temporal region, touch processing

## Abstract

The mother’s attunement to her infant’s emotional needs influences her use of touching behaviors during mother–infant interactions. Moreover, maternal touch appears to modulate infants’ physiological responses to affective touch. However, little is known about the impact of maternal sensitivity on infants’ touch processing at a brain level. This study explored the association between maternal sensitivity when infants (*N* = 24) were 7 months old and their patterns of cortical activation to touch at 12 months. Brain activation was measured using functional near-infrared spectroscopy. Changes in oxy-hemoglobin (HbO_2_) and deoxy-hemoglobin (HHb) concentrations were measured in the left somatosensory cortex and right temporal cortex while infants received two types of tactile stimulation—affective and discriminative touch. Results showed that a lower maternal sensitivity was associated with a higher HbO_2_ response for discriminative touch over the temporal region. Additionally, infants of less sensitive mothers tended to present a higher response in HbO_2_ for affective touch over the somatosensory region. These findings suggest that less sensitive interactions might result in a lower exposure to maternal touch, which can be further related to infants’ neural processing of touch.

Since birth, touch is used to explore and discriminate stimuli in the surrounding physical environment (e.g. identify objects), but it also contributes to establishing social interactions with others, especially at younger ages (Field, 2010; Morrison *et al.*, 2010). Two distinct dimensions of the tactile experience have been described in the literature—discriminative and affective touch. Discriminative touch is mediated by myelinated Aβ fibers, which respond to faster tactile stimulation and allow the detection of changes in stimulus properties (e.g. pressure, vibration, texture and shape), thus supporting the rapid processing and exploration of the physical environment (McGlone *et al.*, 2007, 2014). In turn, affective touch, which is involved in valence encoding (pleasantness/unpleasantness of a given stimuli), requires a different type of afferent—C-tactile (CT) fibers. CT afferents have been identified in the hairy skin, being apparently absent in glabrous skin (e.g. palm of the hand) (Vallbo *et al.*, 1999; McGlone *et al.*, 2014). These are unmyelinated fibers that seem to optimally respond to gentle, slow stroking (1–10 cm/s) (Olausson *et al.*, 2002, 2010; Löken *et al.*, 2009; Ackerley *et al.*, 2014) at skin-like temperature (Ackerley *et al.*, 2014), such as caresses typically occurring in mother–infant interactions (Löken *et al.*, 2009; Olausson *et al.*, 2010; Cascio *et al.*, 2019). Furthermore, empirical evidence points out that stroking delivered at this speed range (1–10 cm/s) is perceived as the most pleasant, when compared to stroking applied at slower or faster paces (e.g. Löken *et al.*, 2009; Essick *et al.*, 2010; Croy *et al.*, 2019).

Each dimension of the tactile experience seems to activate shared and relatively specific neural networks. Prior research with adults has shown that discriminative fibers mainly project to the somatosensory cortices (McGlone *et al.*, 2002; Morrison, 2016), whereas affective touch (slow strokes targeting regions with CT fibers) seems to additionally recruit areas of the so-called ‘social brain’ (Frith, 2007; Adolphs, 2009). More specifically, neuroimaging studies reported consistent patterns of cortical activation in areas such as the insula, medial prefrontal cortex and posterior superior temporal sulcus (pSTS), along with the primary (SI) and secondary (SII) somatosensory cortices, in response to gentle stroking (e.g. Gordon *et al.*, 2013; Voos *et al.*, 2013; Bennett *et al.*, 2014; Björnsdotter *et al.*, 2014; Morrison, 2016).

In addition, a set of studies suggest that the activation of social areas of the brain to affective touch occurs at younger ages—although the precise timing of its emergence is still under examination. In this regard, a functional magnetic resonance imaging (fMRI) study with children and adolescents (5–17 years) demonstrated significant activation in the insula and temporal region, including the pSTS, following soft stroking with a brush on the hairy skin of the arm, compared to stimulation in the palm of the hand (Björnsdotter *et al.*, 2014; Kaiser *et al.*, 2016). In another study using functional near-infrared spectroscopy (fNIRS), infants aged 5 months (Pirazzoli *et al.*, 2019) showed no selective cortical activation in the pSTS region in response to affective (human touch strokes) *vs* non-affective touch (cold spoon strokes) delivered to the infants’ upper arm. Miguel *et al.* (2019) also found a lack of activation in this brain region when comparing affective touch (soft brush strokes) and discriminative touch (light taping with a wooden block) in the infants’ forearm at 7 months (fNIRS study). However, at 12 months of age, these authors found that pSTS was recruited in response to affective touch, similarly to older children and adults (Miguel *et al.*, 2018). In contrast, two other studies suggest that infants as young as 1 and 2 months already recruit social brain regions to process affective touch. Using diffuse optical tomography, Jönsson *et al.* (2018) found that slow stroking (*vs* fast stroking) of the infant’s forearm, using a soft brush, generated significant activation in the insular cortex and temporal lobe of 2-month-olds. Similarly, Tuulari *et al.* (2019) found significant activations in the posterior insular cortex of newborns in response to slow brushing applied to infants’ right anterior shin (fMRI study).

Overall, the abovementioned studies used a bottom-up processing approach, based on stimulation in CT innervated areas. However, the individual’s responsiveness to affective touch is likely influenced by several contextual and relational factors (Ellingsen *et al.*, 2016; Cascio *et al.*, 2019). These factors might not only modulate the neural responses to touch but the resulting attributions of hedonic value and behavioral responses. For example, studies with adults have shown that pleasantness ratings of affective touch are influenced by the degree of exposure to touch in everyday social interactions (Sailer and Ackerley, 2019), as well as the attractiveness (Novembre *et al.*, 2020) and the identity (Gazzola *et al.*, 2012) of the person perceived to convey the touch. Interestingly, the work by Gazzola *et al.* (2012) also found that the visual identity of the caresser modulated the response to affective touch in the primary somatosensory cortex. Moreover, a very recent study also pointed to the importance of relational factors for the early processing of affective touch in infants. Aguirre *et al.* (2019) found heart rate deceleration in 9-month-old infants in response to CT-optimal stroking (brush stroking at 3 cm/s) only when the parent (*vs* an unfamiliar experimenter) was perceived as the source of touch. These results warrant further exploration of the role of early social experiences in modulating the processing of affective touch.

It is widely documented that tactile behavior is a key component in parent–child interactions throughout the first year of life (Ferber *et al.*, 2008; Jean *et al.*, 2009), with mothers spending on average 65% of the time touching their infants (Stack and Muir, 1990). Additionally, empirical evidence suggests variation in frequency and quality of early tactile experiences during caregiving. For example, research with depressed mothers has shown that they touch less often and engage in less positive types of touching behaviors (e.g. gentle stroking) during mother–infant interactions (Malphurs *et al.*, 1996; Mantis *et al.*, 2019). Additionally, mothers showing more intrusive verbal and non-verbal behaviors also demonstrated more negative touching (e.g. rough poking) during interactions with their 3-month-old infants, who, in turn, exhibited less positive behavioral states (e.g. smiling) (Malphurs *et al.*, 1996). Another work, exploring the relation between maternal mind-mindedness and the use of tactile behaviors at 12 months, found that the frequency of mothers’ verbal comments that were non-attuned to their infants’ mental states (e.g. feelings, thoughts and desires) predicted maternal touch behaviors non-contingent with the infants’ emotional state and needs (Crucianelli *et al.*, 2019).

Thus, less frequent, more negative and non-contingent touching may reflect disturbances in maternal sensitivity to the infant’s signals and needs. Sensitivity relates to the caregiver’s ability to acknowledge the infant’s needs and communications, interpret them correctly and provide a contingent and appropriate response (Grossmann *et al.*, 2013), therefore playing an important role in the development of attachment security (e.g. De Wolff and Van Ijzendoorn, 1997). Indeed, maternal touch becomes an important emotional input to regulate infants’ affective states and to physically express protection and affection, which is crucial for the formation of affective bonds with the caregiver. In this regard, 4-month-old infants from dyads characterized by higher-touch dysregulation are more likely to develop future disorganized attachment patterns (Beebe and Steele, 2013). Similarly, Weiss *et al.* (2000) observed that securely attached infants received more nurturing and tender touch (e.g. stroking and kissing) from their mothers. In turn, neuroimaging findings show that 5-year-old children exposed more frequently to maternal touch (presumed to occur more often in the context of sensitive parent–infant interactions) revealed stronger resting-state activity and connectivity in social areas of the brain, particularly in the right pSTS (Brauer *et al.*, 2016).

The literature supports the idea that the quality of early parent–infant interactions may shape the individual’s behavioral and neural responses to tactile stimulation. To the best of our knowledge, no prior study explored the association between maternal sensitivity and infants’ neural processing of touch in the first year of life. Thus, using fNIRS, we aimed to investigate if the history of early caregiving experiences, indexed by maternal sensitivity at 7 months of age, impacts the infant’s cortical responses to touch at 12 months of age. This design is driven by the fact that, by 7 months of age, the use of touch in parent–infant interactions seems to be already predictive of distinct attachment styles. However, a differential brain response to affective and discriminative touch seems to occur only at 12 months, using fNIRS (Miguel *et al.*, 2018). Therefore, in the present study, we followed infants longitudinally to understand if maternal sensitivity was associated with the hemodynamic responses to discriminative and affective touch. We hypothesize that infants exposed to greater maternal sensitivity—more likely to receive frequent and positive touch that is more contingent to their needs and signals—will display a stronger neural response to both types of touch—affective and discriminative—particularly a greater activation in temporal regions of the brain in response to affective touch.

## Methods

### Participants

Participants were 24 infants (13 male, 54%) and their mothers, who were taking part in a larger longitudinal investigation at CIPsi, University of Minho (Miguel *et al.*, 2018). All infants were full-term, except for one infant born preterm but presenting adequate weight at birth (3132 g). Assessments were conducted at 7 (*M* = 7.62, s.d. = 0.37) and 12 months of age (*M* = 12.89, s.d. = 0.31). This study includes participants with complete data at both time points. Initially, 45 infants were assessed with fNIRS at 12 months, but 21 infants were excluded for several reasons: fussiness (*n* = 4), motion artifact (*n* = 7), the minimum number of eligible trials was not obtained (*n* = 7), experimental error (*n* = 2) and no mother–infant interaction data (*n* = 1). Our attrition rate is within the range presented by other infant fNIRS studies (Lloyd-Fox *et al.*, 2010). Mothers’ age ranged from 22 to 39 years (*M* = 33.08, s.d. = 4.12), and the majority had high education qualifications (*n* = 20, 83%). All participants were Whites, and Portuguese was their spoken language at home.

### Maternal sensitivity

Mother–infant interaction was coded using the Sensitivity vs. Insensitivity scale of Maternal Sensitivity Scales (Ainsworth *et al.*, 1978). The interaction consisted of three episodes of 3 min each and with specific challenges being presented to the dyad: first, mothers played with their infants using some age-appropriate toys as they would normally do at home; then, the dyad played without toys and, finally, the mother was instructed to teach the infant how to play with a challenging toy (considered to be above the infant’s current developmental level). The maternal interactive style was evaluated according to the mother’s degree of attunement (appropriateness and contingency of response) to her infant’s needs and signals (Ainsworth, 1969; Ainsworth *et al.*, 1978). Mothers’ behavior was rated on a 9-point scale, ranging from 1 (‘highly insensitive’) to 9 (‘highly sensitive’), such that higher scores reflected a more positive and sensitive interactive style. A global score was assigned to the entire interaction based on the mother’s interactive behavior across all three episodes. A random sample of 25% of the mother–infant interactions was coded for reliability purpose and inter-observer agreement revealed to be adequate (Intraclass Correlation Coefficient (ICC) = 0.95).

### fNIRS assessment

#### Stimuli.

The experimental protocol included two types of tactile stimulation—affective and discriminative touch—delivered manually to the infant’s right dorsal forearm by one trained experimenter. The affective touch condition consisted of slow strokes (8 cm/s), using a 7 cm wide watercolor brush (Bennett *et al.*, 2014; Kaiser *et al.*, 2016), administered from the proximal to the distal part of the infant’s forearm. In the discriminative stimuli condition, a squared-shape piece of wood 2 × 2 cm (Kida and Shinohara, 2013) was used to apply pressure on the infant’s dorsal forearm, also in a proximal–distal direction. The wood block was applied three times per second at different points of the forearm (around 21–24 stimuli per trial). No stroking movement was included in the discriminative condition, such that only Aβ fibers were stimulated.

**Fig. 1.**
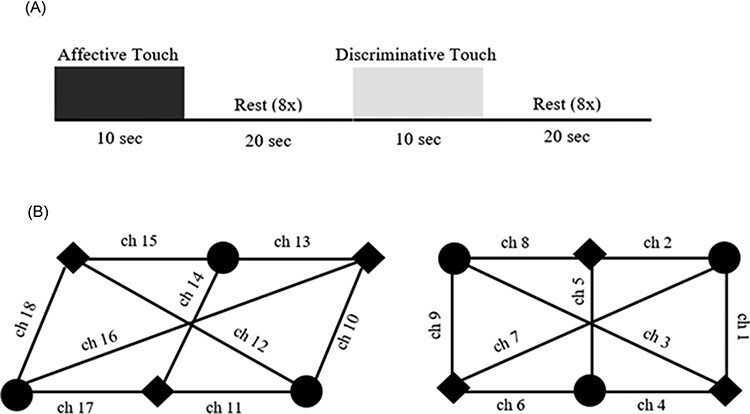
(A) Experimental protocol consisting of two tactile stimulus conditions (affective and discriminative), each stimulus was delivered for 10 s, followed by 20 s of rest, across eight trials. Two blocks were presented per experimental condition. (B) fNIRS layout showing the location of 18 channels: 9 placed over right temporal region (left panel) and 9 placed over the left (contralateral) primary somatosensory cortex (right panel). The circles represent sources and the squares correspond to detectors. For further information on fNIRS headgear, please see Miguel et al. (2018).

The experimental protocol was presented in a within-subject block design, divided into two alternating blocks per condition (affective and discriminative). Each block consisted of eight trials, composed of 10 s of tactile stimulation followed by a 20 s baseline (rest) period each (Figure 1). In turn, the baseline stimuli consisted of a silent cartoon movie (Czech cartoon Krtecek) that played continuously throughout the entire procedure (Fairhurst *et al.*, 2014).

#### NIRS probe.

Hemodynamic activity in response to tactile stimuli was measured with the UCL-fNIRS topography system (Everdell *et al.*, 2005) composed of 12 sources and 6 detectors, using two continuous wavelengths of source light at 780 and 850 nm. Data were sampled every 100 ms (10 Hz) (for further details of fNIRS methodology, please see Lloyd-Fox *et al.*, 2010).

An array with 18 channels was used to collect hemodynamic response. Nine channels covered the left (contralateral) primary somatosensory cortex, whereas the remaining nine were placed over the right temporal region (Figure 1), since previous studies have shown that this latter brain area is involved in the processing of affective touch (e.g. Voos *et al.*, 2013; Bennett *et al.*, 2014). Source-detector separation was between 20 and 25 mm in the somatosensory region and 22 mm in the temporal region. Additional four channels (channels 3, 7, 12 and 16) that crossed the middle of the array were 45 mm.

Infants wore a customized NIRS headgear (Easy Cap) with reference to the 10-5 system coordinates (Jurcak *et al.*, 2007). Prior to the beginning of the experimental protocol, infants’ head circumference (*M* = 46.45 cm, s.d. = 1.26) and nasion–inion (*M* = 29.96 cm, s.d. = 1.29) measurements were taken for cap adjustments. The cap was positioned centrally in the top of Cz, with the channel 11 (correspondent to TP8) placed above the peri-auricular point.

#### NIRS data processing.

Infant behavior during the fNIRS session was videotaped and, subsequently, coded offline by a trained observer, who was blind to the inclusion criteria. Participants were included in the analyses if they obtained a minimum of three trials of good quality (Lloyd-Fox *et al.*, 2015; Miguel *et al.*, 2018; Lloyd-Fox *et al.,* 2019). A trial was considered acceptable if (i) no movements of the right arm were observed during the stimulus administration and (ii) the infant did not look at and/or touch the experimenter or the mother while the stimulus was being delivered. Trials were also excluded if infants moved their right arm during baseline period preceding or following the trial. Infants contributed on average with 6.8 trials (s.d. = 2.1) in the affective condition (range 4–13) and 6.4 trials (s.d. = 2.5) in the discriminative condition (range 3–12). Finally, the experimental conditions did not differ on the number of valid trials.

The NIRS data were processed using HOMER2 (MGH—Martinos Center for Biomedical Imaging, Boston, MA, USA), a MATLAB software package (The MathWorks, Inc., Natick, MA, USA), for measures of oxy-hemoglobin (HbO_2_) and deoxy-hemoglobin (HHb) concentration change (µmol). Only valid trials for each experimental condition (affective and discriminative) were retained in HOMER2 for data processing. The attenuated light intensities, measured by the detecting optodes, were converted to optical density units and assessed for motion artifact using principal component analysis set at 0.9. (Cooper *et al.*, 2012). Data were low-pass filtered at 0.5 Hz (Lloyd-Fox *et al.*, 2015), and changes in concentration of the hemoglobin chromophore were calculated in accordance to the modified Beer–Lambert Law (Delpy *et al.*, 1988) and assuming a path length factor of 5 for both wavelengths (Duncan *et al.*, 1995). Traces were segmented into 30 s epochs around the trigger stimulus for each trial with each epoch, starting 2 s prior to each stimulus presentation. Baseline correction corresponded to the mean HbO_2_/HHb values from −2 to 0, as in previous fNIRS studies (Ravicz *et al.*, 2015). This preliminary analysis also led us to conclude that the long channels (channels 3, 7, 12 and 16) resulted in noisy data and, for that reason, were excluded from subsequent analyses for all the subjects.

For each participant, the hemodynamic response was averaged across all valid trials, representing the mean change in HbO_2_ and HHb concentration for each channel and type of stimulus. Then, for each participant and each channel, we extracted the peak amplitude of HbO_2_ (maximum hemoglobin increase) and HHb (maximum hemoglobin decrease) between 10 and 20 s in response to each experimental condition to be used as the dependent variable in subsequent analyses (Miguel *et al.*, 2018, 2019). Due to a greater signal-to-noise ratio and similarly to previous fNIRS studies, we only used HbO_2_ signal for the remaining analyses (Lloyd-Fox *et al*., 2010, 2017, 2019).

### Procedure

This study was approved by the ethics board of the university that coordinated the study. The participating families were recruited from local parenting classes, social networks and daycare centers. Individual sessions were carried out when infants were 7 and 12 monthsold, according to the mother’s availability and at a time of the day the infant would be in quiet and alert state. Mothers received information regarding the objectives of the study and all the procedures to be conducted, after which they signed the consent form authorizing their own and their infants’ participation in the study.

At the 7-month assessment, mothers were instructed to play with their infant, for 9 min in total, attending to three distinct scenarios: play freely with toys, play without toys and play with a challenging toy. At 12 months, infants completed a second visit to evaluate the neural processing to tactile stimulation using fNIRS. Prior to the experiment, the researcher took head measurements and placed the cap, while the infant was seated on the mother’s lap. Then, the infant was moved to a baby seat (Jellymom baby chair), in order to prevent any physical contact with the mother. The dyad sat in front of a computer screen, set up at approximately 70 cm distance, watching a silent movie (Czech cartoon Krtecek as in Fairhurst *et al.*, 2014). Mothers were instructed to refrain from any interaction with the infant, except in case the infant became fussy. In turn, the researcher was positioned at right behind the dyad to deliver the tactile stimulation.

During the experiment, the researcher also avoided any contact with the infant and would only intervene to redirect the infant’s attention to the screen. Trials were rejected if the researcher interacted with the infant during trial and/ or baseline period. The NIRS assessment ended when the four blocks of stimuli were presented or if the infant was hard to soothe. The entire session took place in a dimmed-light room, minimizing light interference. Both sessions were recorded for subsequent behavioral coding.

### Statistical analysis

Hemodynamic peak amplitude (i.e. maximum/minimum hemoglobin concentration) change in HbO_2_ and HHb was first assessed in relation to the baseline, using paired *t*-tests. Problems of multiple comparisons were solved by applying the false discovery rate correction (Benjamini and Hochberg, 1995). In addition, correlation analyses were conducted to explore the association between maternal sensitivity and peak amplitude of HbO_2_ for those channels with significant activation. Statistical tests were computed using IBM SPSS Statistics 21.0 (IBM Corporation, Armonk, NY, USA).

## Results

Results showed a significant activation for all channels placed over the somatosensory and temporal regions, in response to affective and discriminative touch (all *P*-values ≤0.022). The Supplementary Materials present the paired *t*-test results for each channel-by-channel contrast of HbO_2_ and HHb peak amplitude against their baseline (see Supplementary Table S1), as well as figures with the time course of hemodynamic response for the two touch conditions, in each channel. Regarding maternal behavior, sensitivity scores ranged from 3 to 9 (*M* = 6.2, s.d. = 1.7), reflecting an insensitive to a highly sensitive style of interaction.

**Fig. 2.**
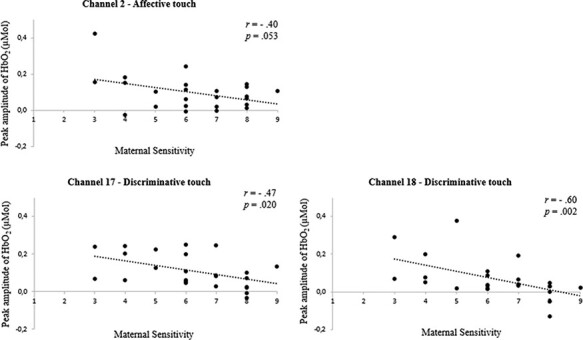
Association between maternal sensitivity and peak amplitude of oxy-hemoglobin (HbO_2_) in channel 2 (somatosensory region) in response to affective stimuli and channels 17 and 18 (temporal region) in response to discriminative stimuli.

Correlation analysis showed that, for affective touch, maternal sensitivity was unrelated to peak amplitude of HbO_2_ in the channels located over the temporal region. For the channels placed over the somatosensory cortex, a marginally significant association was found in channel 2, *r* =−0.40, *P* = 0.053, such that infants whose mothers were less sensitive tended to present a higher response peak in HbO_2_ to the affective touch condition (Figure 2). In turn, discriminative touch yielded no significant association between HbO_2_ peak response and maternal sensitivity in channels covering the somatosensory region. However, for channels centered over the temporal area, less sensitive maternal behavior was significantly associated with infants’ higher peak amplitude of HbO_2_ in channel 17, *r* =−0.47, *P* = 0.020, and channel 18, *r_s_* =−0.60, *P* = 0.002 (Figure 2).

## Discussion

The present study examined the association between maternal sensitivity at 7 months and infants’ neural response to discriminative and affective touch at 12 months of age. Contrary to our hypothesis, results showed that lower maternal sensitivity was associated with greater increase of HbO_2_ in response to affective touch over the somatosensory cortex. Moreover, for discriminative touch, an increase in HbO_2_ in channels over the temporal region was associated with less sensitive maternal interactions.

Previous behavioral evidence suggests that less optimal maternal interactions are likely to result in reduced frequency of tactile stimulation and more non-contingent/negative touching behaviors (Malphurs *et al.*, 1996; Crucianelli *et al.*, 2019; Mantis *et al.*, 2019). Thus, we hypothesize that infants whose mothers were less sensitive, and, therefore, more likely to be exposed to impoverished and dysregulated tactile experiences may have perceived the light touch delivered in the task as more novel, thus engaging in increased neural processing of the stimulus’ properties and significance. Also, infants seem to employ more active self-touching behaviors to regulate their affective states when their mothers are perceived as physically and emotionally unavailable (Moszkowski *et al.*, 2009). Thus, a history of exposure to less sensitive maternal interactions, in which maternal touch might be absent, inconsistent, non-contingent and non-attuned to the infants’ cues and signals, may result in greater reactivity when the tactile stimulus derives from an external source (e.g. the mother) *vs* the own infant (self-touch).

In addition, previous research suggests that familiarity with a specific type of stimulus may lead to diminished response. For example, in an fMRI study in adults, greater response in the right temporoparietal junction, with extended activation in the superior temporal gyrus, revealed sensitivity to stimulus novelty across multiple sensory modalities (visual, auditory and tactile) (Downar *et al.*, 2002). In another work with event-related potentials, De Haan *et al.* (2004) observed that 7-month-old infants whose mothers were high in positive affect showed increased reactivity (longer looking time and larger Nc amplitude) to fearful (potential novel expression) compared to happy facial expressions (probably more familiar). In addition, 3-month-old infants of non-depressed mothers looked longer at sad visual combined with auditory stimuli compared to infants of depressed mothers (Field *et al.*, 1998). Similarly, Striano *et al.* (2002) also found that, at 6 months, infants whose mothers reported more depressive symptoms gazed at them for longer periods when they smiled. Thus, this may explain the novel findings of an association between lower maternal sensitivity and increased touch processing in the temporal region.

The fact that a stronger pattern of association with maternal interactive behavior was observed for discriminative (*vs* affective) touch may reflect some infants’ difficulties in perceiving sensory information and distinguishing subtle aspects of tactile stimulation (light tapping *vs* slow stroking), as a result of poorer interpersonal affective touch experiences during critical periods in early development. In this respect, Sailer and Ackerley (2019) suggest that the degree of exposure to tactile experiences shapes the perception and processing of affective touch. The authors found that adults rarely receiving touch in everyday social situations were less able to differentiate between different stroking velocities, by rating touch delivered at CT-optimal velocity as less pleasant, when compared to adults reporting frequent touch exposure. Also along this line are the findings by Spitoni *et al.* (2020), in which adults with a disorganized (*vs* organized) attachment style find non-CT targeted touch more pleasant than caress-like CT touch. Furthermore, these individuals show an activation in the right limbic/paralimbic cortex, especially in response to non-affective touch. The authors suggested that the recruitment of these brain areas represents a key node that supports an atypical alert state to touch in adults with disorganized attachment.

In future studies, it would be interesting to examine whether, in infancy, maternal sensitivity may contribute to a more fine-grained discrimination of the affective aspects of touch by the infant, for example, if it was applied in an interpersonal condition (human hand) or an impersonal condition (delivered by objects). Most of the infant neuroimaging studies available administered object-mediated touch (for an exception, see Pirazzoli *et al.*, 2019). However, the precise timing of the emergence and the potential impact of maternal sensitivity on such an effect in infants remain to be explored. In this case, a longitudinal approach may enlighten the impact maternal behavior may have on the infant’s processing of affective aspects of touch through time.

The present study has limitations that merit attention in future research. The major limitation regards the absence of a direct measure of maternal touch. Although this study aimed to address the links between infant touch processing and a more general dimension of maternal behavior (i.e. maternal sensitivity), future research may assess whether this relationship is mediated by aspects such as the frequency, type and contingency of maternal touch during mother–infant interactions. In addition, although the range of maternal sensitivity ratings was quite broad, our sample was mostly composed of low-risk dyads, as none of the participating mothers was classified as highly insensitive and the average score reflected a sensitive interactive style. Further work incorporating higher heterogeneity on maternal sensitive behavior may better capture the effect of more negative and intrusive parental interactions on infants’ brain response to affective touch. In this line, other maternal characteristics and behaviors may provide additional insights on the influence of relational factors on the neural processing of touch during the first year of life. Relevant candidates are mothers’ positive affect and own attitudes toward social touch. Regarding the latter, two previous studies have shown that greater maternal preference for social touch is related to infants’ larger decreases in heart rate in response to CT-targeted touch (Fairhurst *et al.*, 2014; Aguirre *et al.*, 2019).

Future studies may also benefit from including a measure of infant’s cardiac activity, which has been shown to decelerate in response to CT-optimal velocity strokes (e.g. Aguirre *et al.*, 2019). This may clarify whether and how physiological and neural systems interact early on in development to process affective touch. Future studies may also explore the contribution of maternal behavior (e.g. sensitivity and touching behaviors) toward the development of specific neural signatures of affective and non-affective touch in infancy. Lastly, the lack of previous studies on this specific topic, coupled with results not confirming our initial hypotheses, led to a certain degree of speculation in our explanations. Hopefully, future studies may help to clarify the proposal advanced here.

In conclusion, our findings extend previous literature on the effect of maternal behavior on infants’ processing of tactile experiences, particularly the contribution of observed maternal sensitive interactions to the child’s neural responses to gentle touch. In turn, understanding how early parental behaviors influence infants’ processing of affective touch may inform early intervention programs tailored to promote nurturing parent–infant interactions and, ultimately, foster optimal infant development.

## Supplementary Material

nsab069_Supp

## Data Availability

The data that support the findings of this study are available from the corresponding author upon reasonable request.
