# Supplementary material for: Maternal sensitivity and infant neural response to touch: an fNIRS study
Source: Soc Cogn Affect Neurosci. 2021 Jun 18;16(12):1256–63. doi: 10.1093/scan/nsab069 (PMC8716843; doi:10.1093/scan/nsab069)

Table S1. Channel-by-channel contrast of HbO_2_ and HHb peak amplitude against baseline

|  | Affective touch | | | | | | Discriminative touch | | | | | |
| --- | --- | --- | --- | --- | --- | --- | --- | --- | --- | --- | --- | --- |
|  | HbO_2_ | | | HHb | | | HbO_2_ | | | HHb | | |
|  | *t* | *p* | *d_z_* | *t* | *p* | *d_z_* | *t* | *p* | *d_z_* | *t* | *p* | *d_z_* |
| Somatosensory region |  |  |  |  |  |  |  |  |  |  |  |  |
| Ch 1 | - 5.33 | < .001 | 1.09 | 5.95 | < .001 | 1.21 | - 3.69 | .001 | 0.75 | 4.92 | < .001 | 1.00 |
| Ch 2 | - 4.99 | < .001 | 1.02 | 5.32 | < .001 | 1.09 | - 2.99 | .007 | 0.61 | 3.86 | .001 | 0.79 |
| Ch 4 | - 5.56 | < .001 | 1.13 | 3.79 | .001 | 0.77 | - 4.72 | < .001 | 0.96 | 4.61 | < .001 | 0.94 |
| Ch 5 | - 6.36 | < .001 | 1.30 | 6.43 | < .001 | 1.31 | - 5.00 | < .001 | 1.02 | 4.18 | < .001 | 0.85 |
| Ch 6 | - 4.02 | .001 | 0.82 | 4.65 | < .001 | 0.95 | - 4.12 | < .001 | 0.84 | 3.01 | .006 | 0.61 |
| Ch 8 | - 4.19 | < .001 | 0.86 | 5.40 | < .001 | 1.10 | - 3.37 | .003 | 0.69 | 4.48 | < .001 | 0.91 |
| Ch 9 | - 6.59 | < .001 | 1.35 | 5.91 | < .001 | 1.21 | - 3.87 | .001 | 0.79 | 4.31 | < .001 | 0.88 |
| Temporal region |  |  |  |  |  |  |  |  |  |  |  |  |
| Ch 10 | - 5.22 | < .001 | 1.07 | 4.90 | < .001 | 1.00 | - 3.55 | .002 | 0.72 | 3.28 | .003 | 0.67 |
| Ch 11 | - 3.84 | .001 | 0.78 | 3.40 | .002 | 0.69 | - 2.72 | .012 | 0.56 | 3.16 | .004 | 0.65 |
| Ch 13 | - 4.78 | < .001 | 0.98 | 4.39 | < .001 | 0.90 | - 4.45 | < .001 | 0.91 | 2.47 | .022 | 0.50 |
| Ch 14 | - 5.55 | < .001 | 1.13 | 4.34 | < .001 | 0.89 | - 4.45 | < .001 | 0.91 | 2.54 | .018 | 0.52 |
| Ch 15 | - 5.78 | < .001 | 1.18 | 4.86 | < .001 | 0.99 | - 3.95 | .001 | 0.81 | 2.46 | .022 | 0.50 |
| Ch 17 | - 4.86 | < .001 | 0.99 | 5.38 | < .001 | 1.10 | - 6.19 | < .001 | 1.26 | 9.53 | < .001 | 1.95 |
| Ch 18 | - 5.52 | < .001 | 1.13 | 4.32 | < .001 | 0.88 | - 3.23 | .004 | 0.66 | 5.50 | < .001 | 1.12 |

Ch = channel; HbO_2_ = oxy-hemoglobin; HHb = deoxy-hemoglobin. *d_z_* = effects sizes calculated in accordance to Lakens (2013)^1^.

________________

^1^ Lakens, D. (2013). Calculating and reporting effect sizes to facilitate cumulative science: a practical primer for t-tests and ANOVAs. *Frontiers in Psychology*, *4*, 863. https://doi.org/10.3389/fpsyg.2013.00863

Figure S1. Hemodynamic response to affective touch over the somatosensory and temporal regions


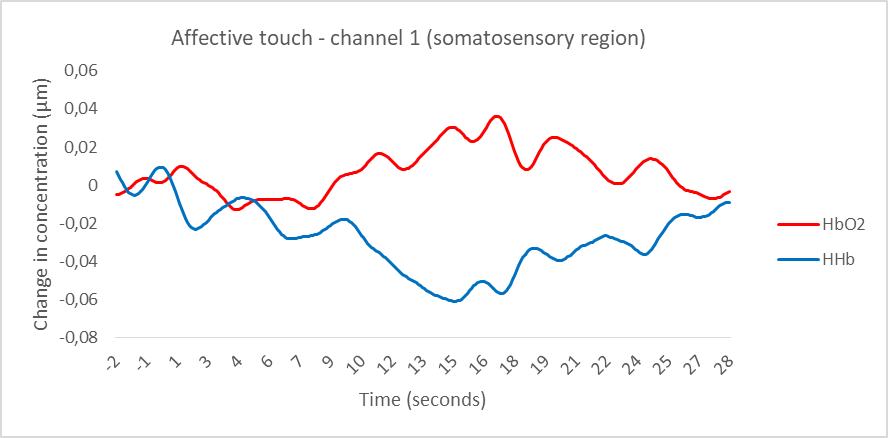

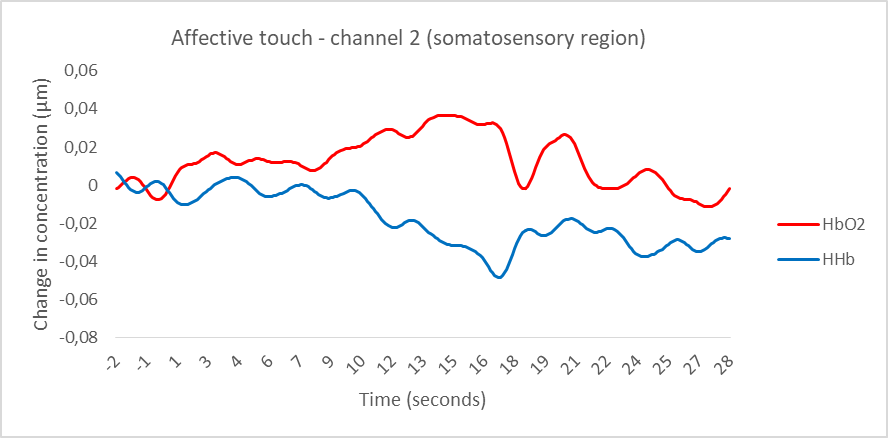


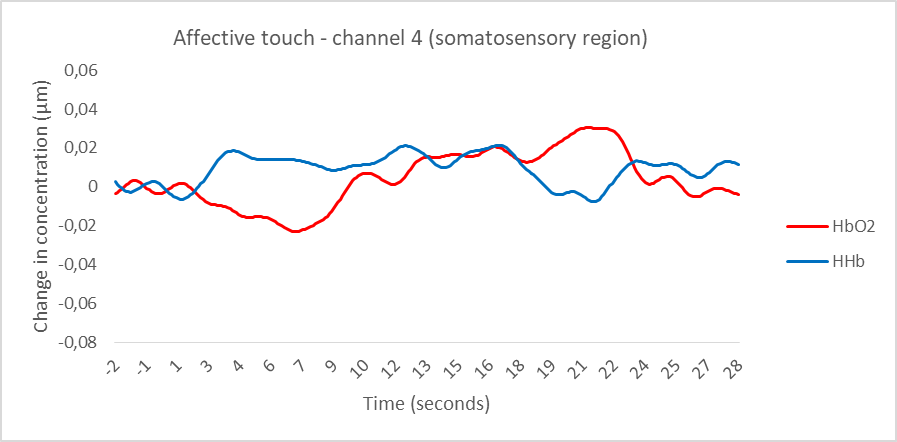


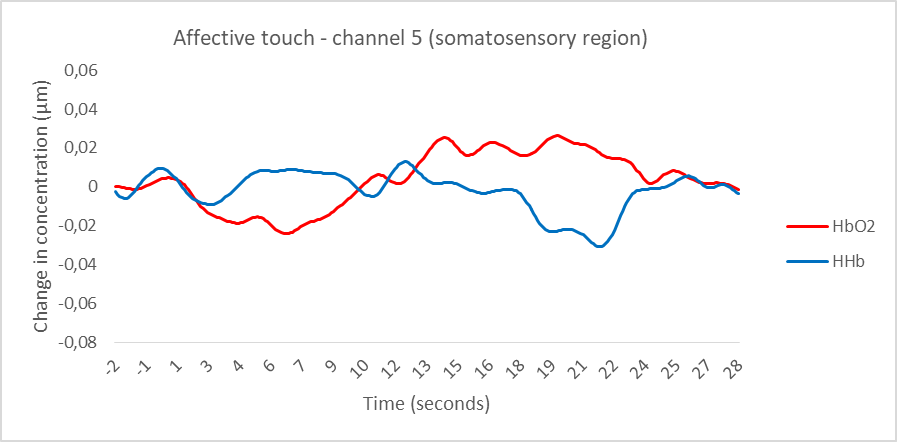


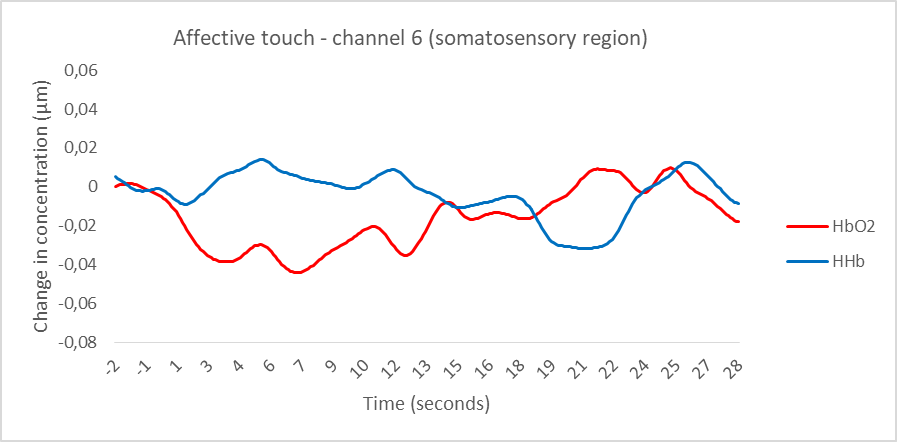


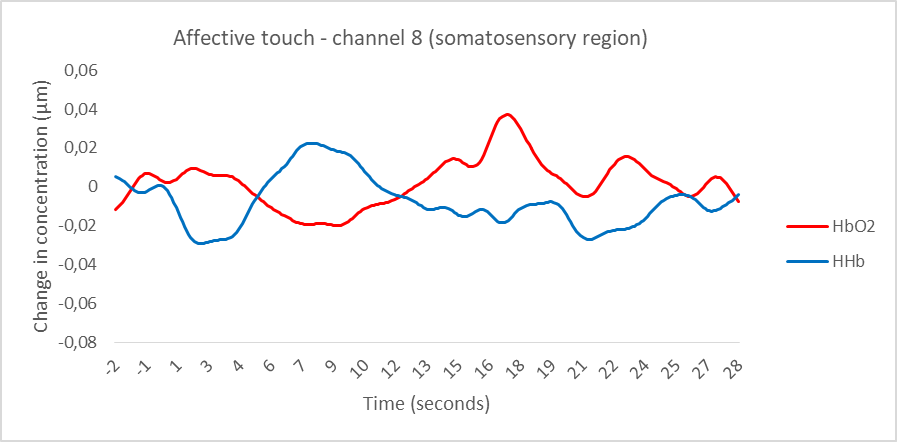


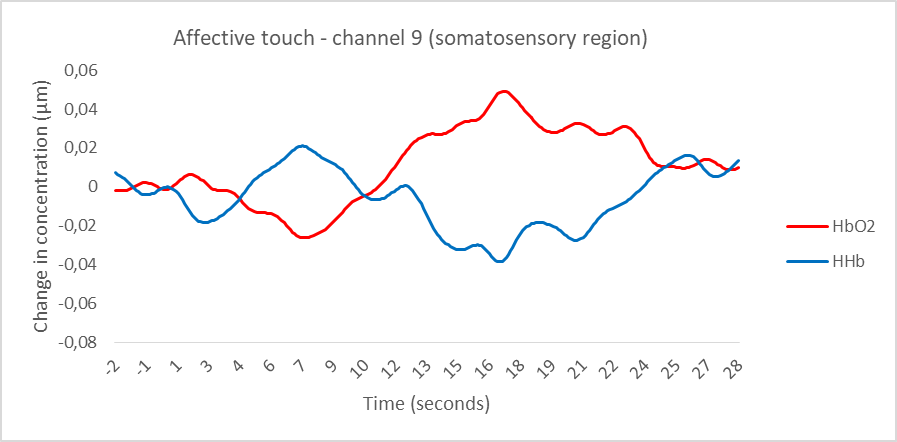


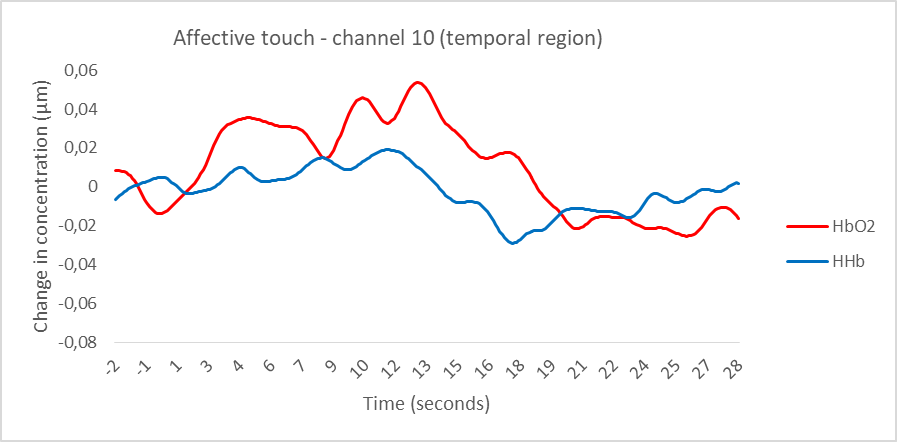


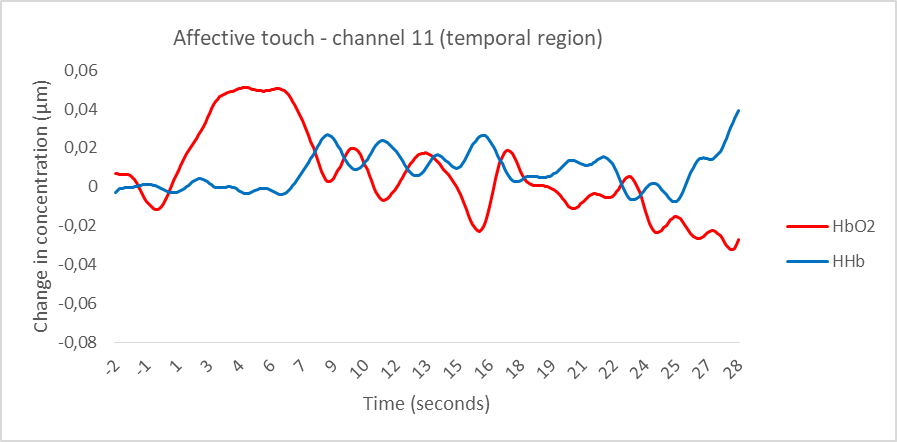


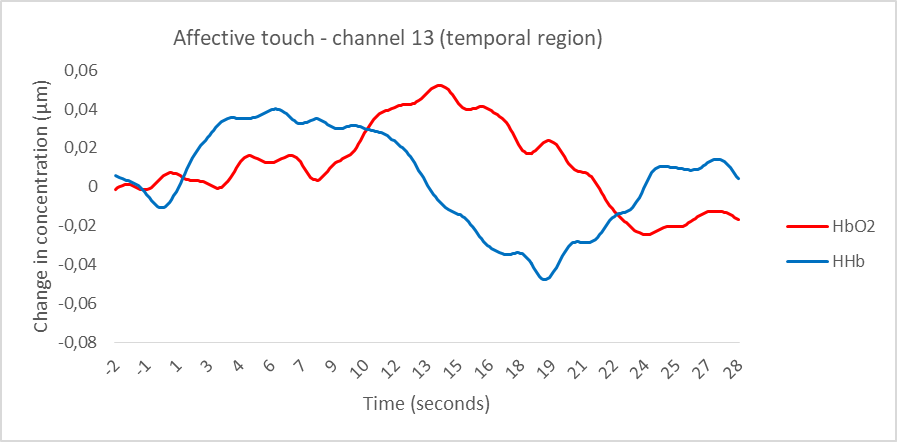


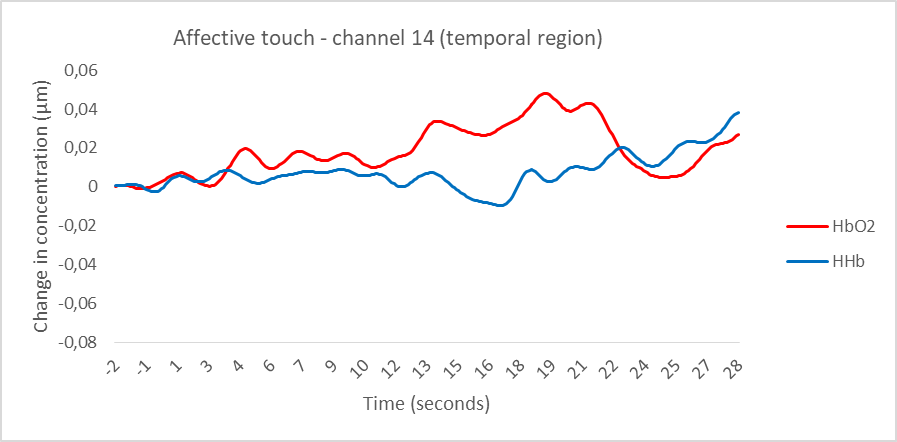


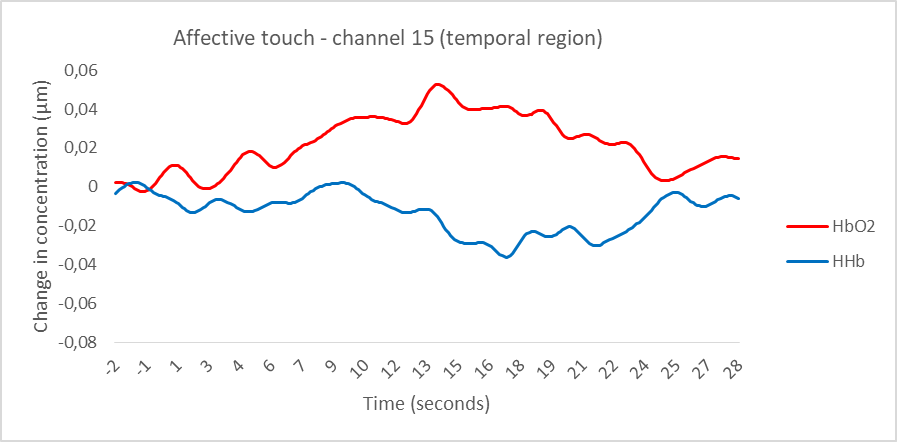


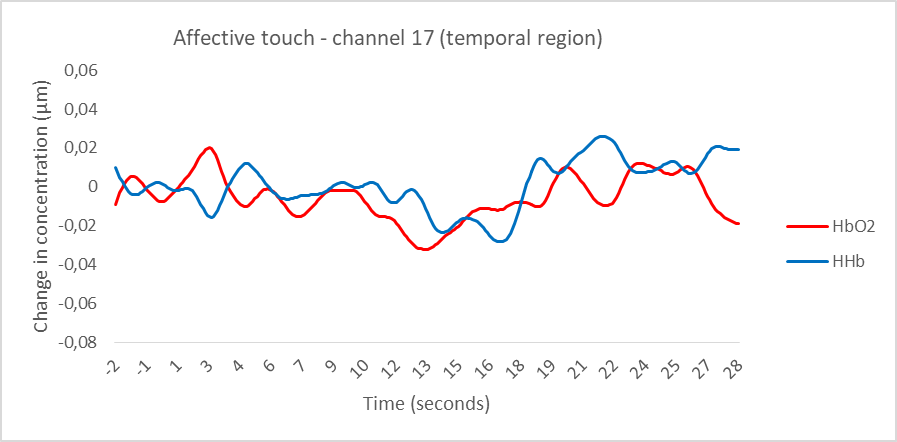


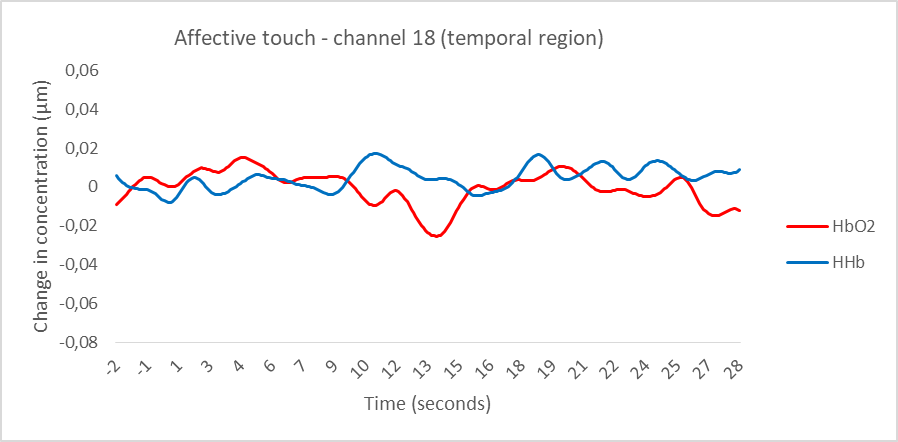


Figure S2. Hemodynamic response to discriminative touch over the somatosensory and temporal regions


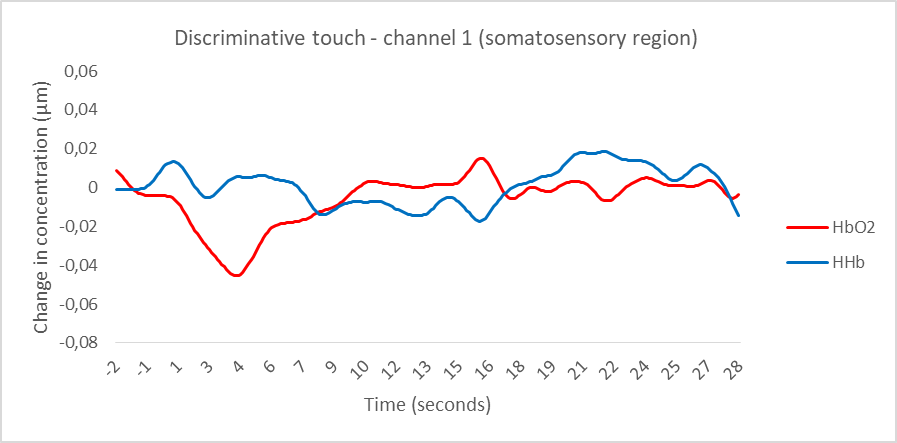

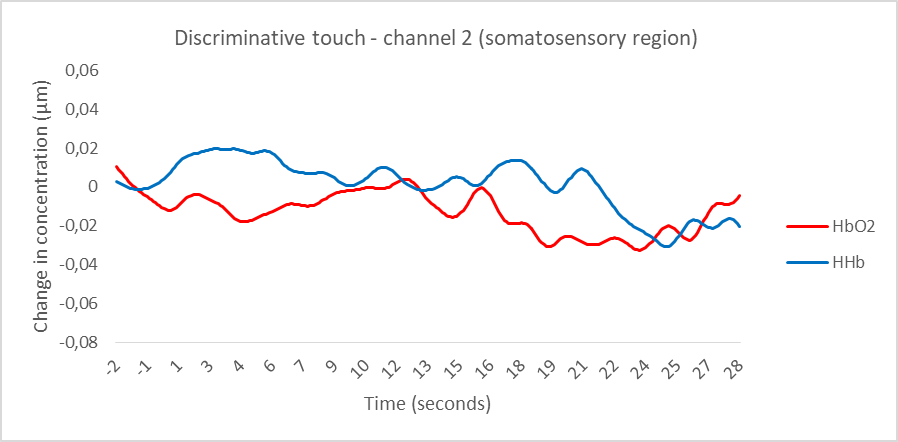


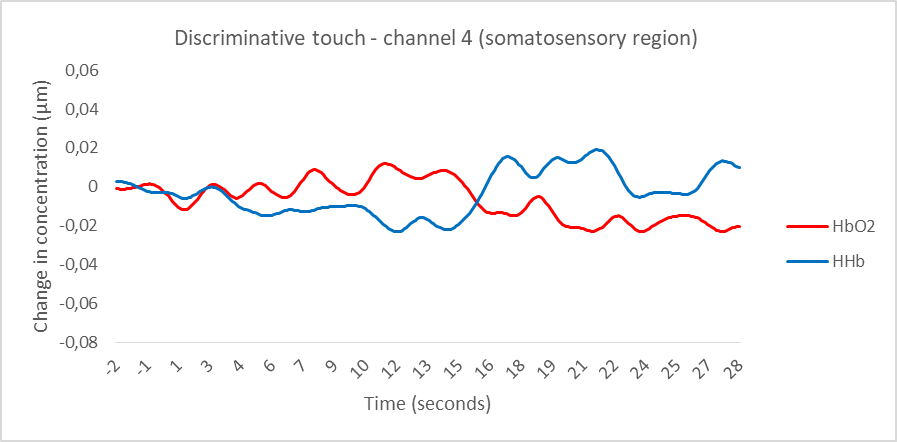


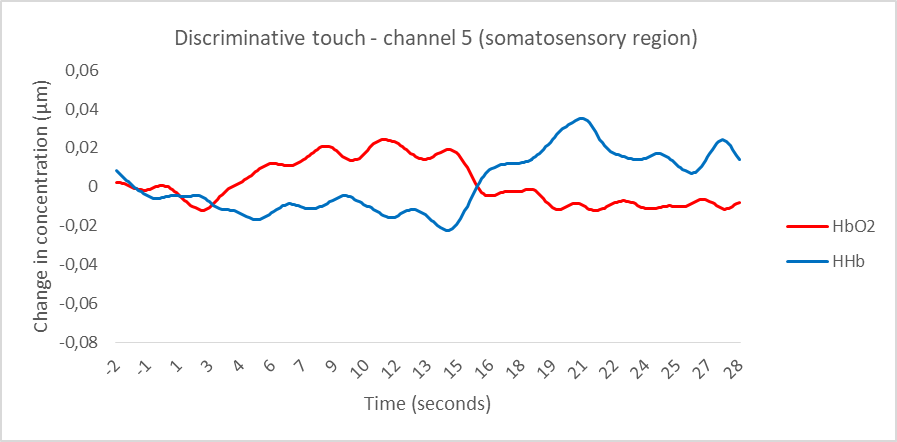


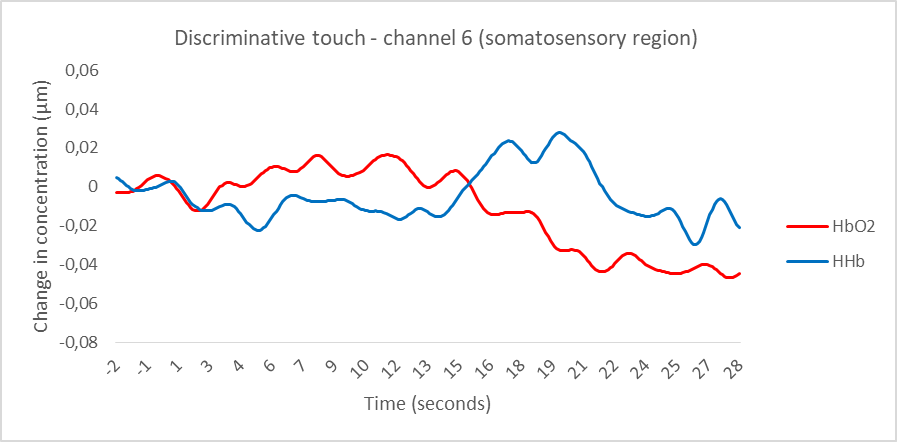


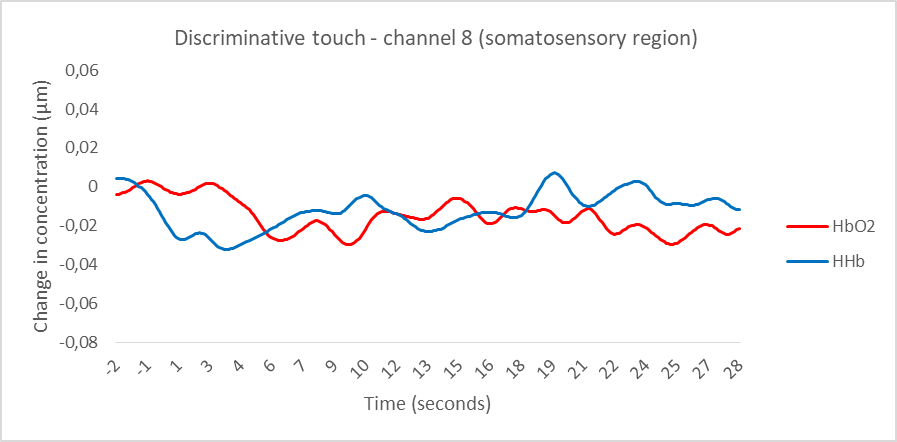


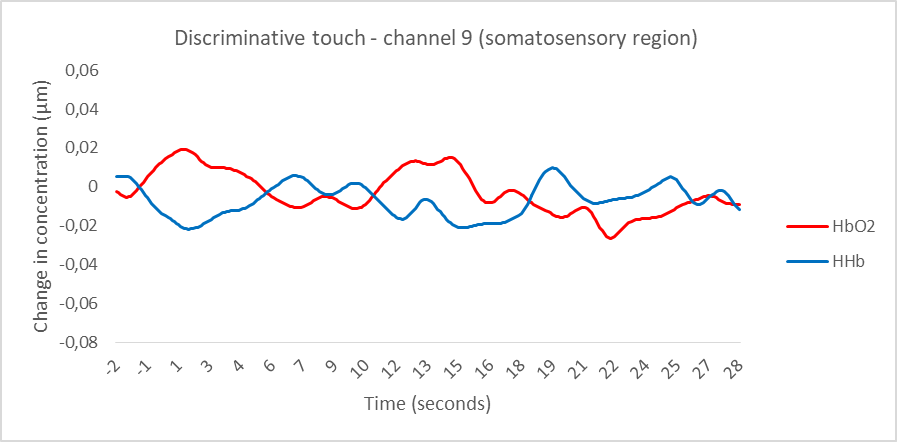


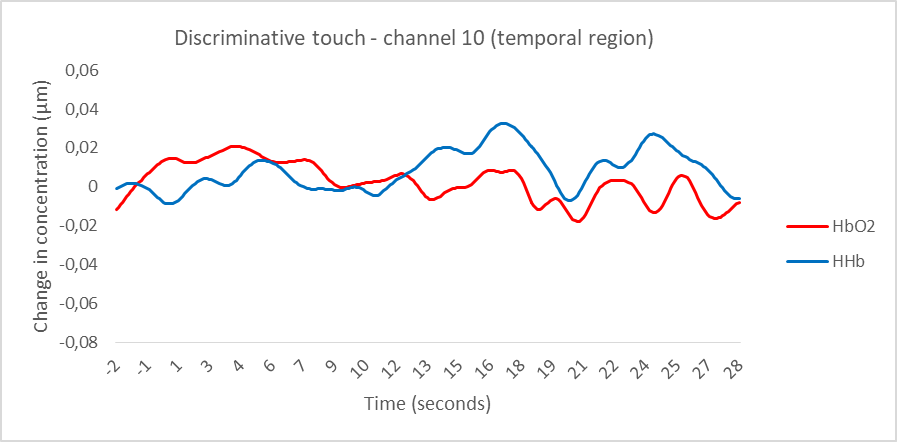


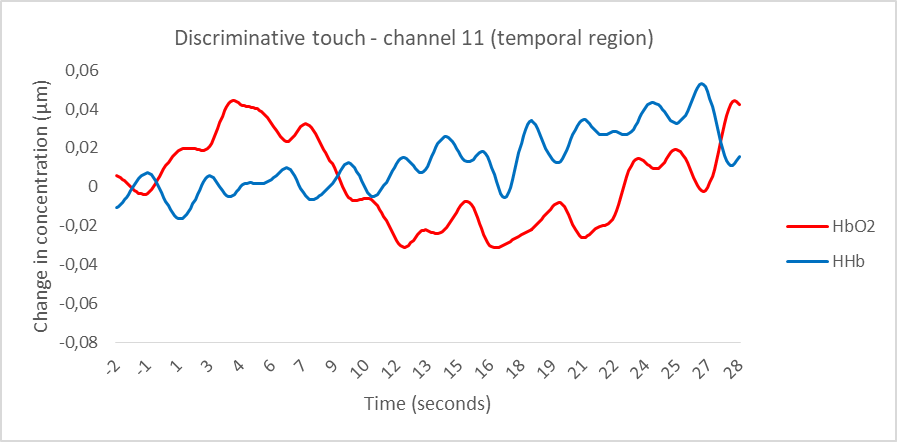


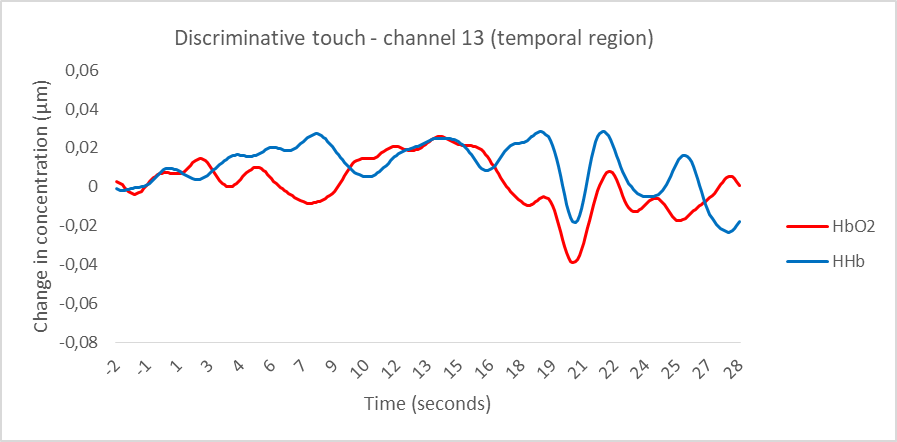


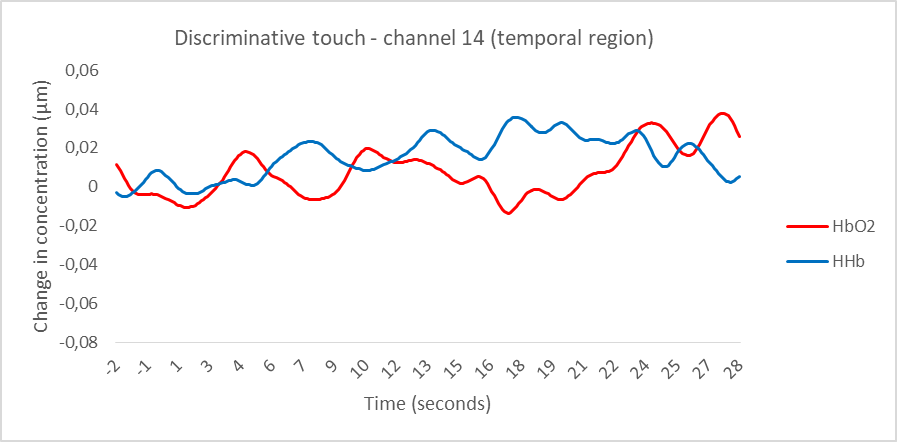


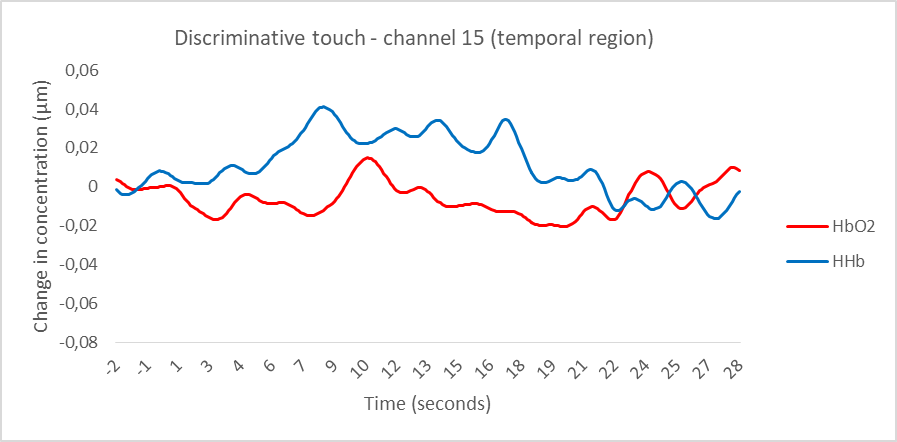


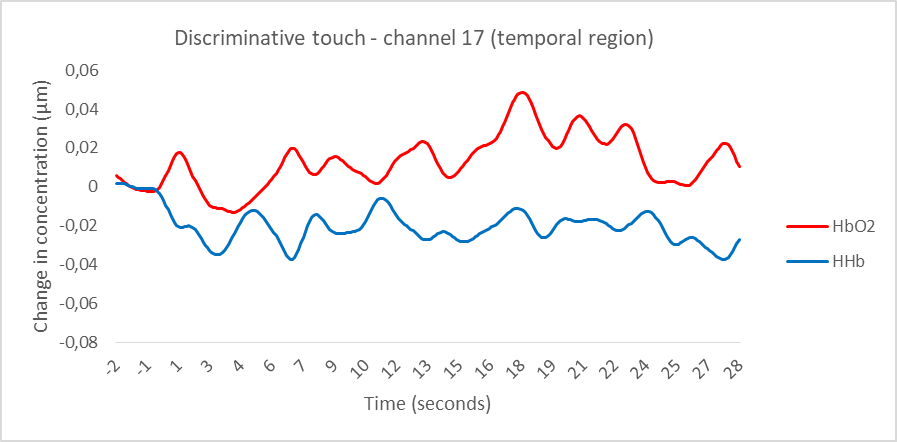


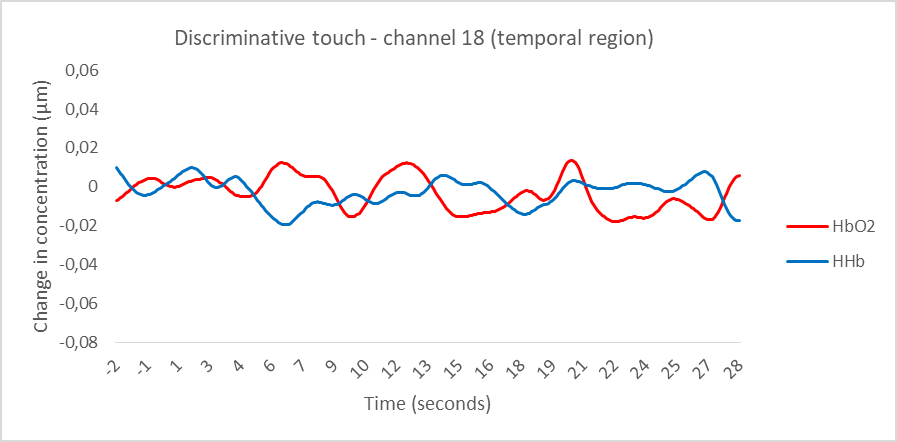

Supplement: nsab069_Supp [file nsab069_supp.zip › scan-21-002-File004.docx]
